# Gene therapy for selected neuromuscular and trinucleotide repeat disorders – An insight to subsume South Asia for multicenter clinical trials

**DOI:** 10.1016/j.ibneur.2023.01.009

**Published:** 2023-01-30

**Authors:** Nalaka Wijekoon, Lakmal Gonawala, Pyara Ratnayake, Darshana Sirisena, Harsha Gunasekara, Athula Dissanayake, Sunethra Senanayake, Ajantha Keshavaraj, Yetrib Hathout, Harry W.M. Steinbusch, Chandra Mohan, Ashwin Dalal, Eric Hoffman, K.Ranil D de Silva

**Affiliations:** a Interdisciplinary Centre for Innovations in Biotechnology and Neuroscience, University of Sri Jayewardenepura, Nugegoda, Sri Lanka; b School for Mental Health and Neuroscience, Faculty of Health, Medicine & Life Sciences, Maastricht University, Maastricht, the Netherlands; c EURON - European Graduate School of Neuroscience, the Netherlands; d Lady Ridgeway Hospital for Children, Colombo, Sri Lanka; e Colombo North Teaching Hospital, Ragama, Sri Lanka; f Sri Jayewardenepura General Hospital, Colombo, Sri Lanka; g Teaching Hospital Karapitiya, Galle, Sri Lanka; h National Hospital of Sri Lanka, Colombo, Sri Lanka; i Teaching Hospital Jaffna, Sri Lanka; j Pharmaceutical Sciences Department, School of Pharmacy and Pharmaceutical Sciences, Binghamton University, New York, USA; k Dept. of Brain & Cognitive Sciences, Daegu Gyeungbuk Institute of Science and Technology (DGIST), Daegu, Republic of Korea; l Department of Bioengineering, University of Houston, Houston, TX, USA; m Diagnostics Division, Center for DNA Fingerprinting and Diagnostics, India; n Institute for Combinatorial Advanced Research and Education (KDU-CARE), General Sir John Kotelawala Defence University, Ratmalana, Sri Lanka

**Keywords:** Indian Sub-continent, Developing Countries, Neurogenetic Disorders, Bio Bank, Duchenne Muscular Dystrophy, Spinal Muscular Atrophy, Spinocerebellar Ataxia, Huntington’s Disease, BMD, Becker muscular dystrophy, DMD, Duchenne muscular dystrophy, EMA, European Medical Agency, EMQN, European Molecular Quality Genetics Network, FDA, U. S. Food and Drug Administration, HD, Huntington’s disease, HIC, High Income Countries, MLPA, Multiplex Ligation Dependent Probe Amplification, SMA, Spinal muscular atrophy, SCA, Spinocerebellar ataxia, UMIC, Upper Middle Income Countries, WTO, World Trade Organization

## Abstract

**Background:**

In this article, the authors discuss how they utilized the genetic mutation data in Sri Lankan Duchenne muscular dystrophy (DMD), Spinal muscular atrophy (SMA), Spinocerebellar ataxia (SCA) and Huntington’s disease (HD) patients and compare the available literature from South Asian countries to identifying potential candidates for available gene therapy for DMD, SMA, SCA and HD patients.

**Methods:**

Rare disease patients (n = 623) with the characteristic clinical findings suspected of HD, SCA, SMA and Muscular Dystrophy were genetically confirmed using Multiplex Ligation Dependent Probe Amplification (MLPA), and single plex PCR. A survey was conducted in the “Wiley database on Gene Therapy Trials Worldwide” to identify DMD, SMA, SCA, and HD gene therapy clinical trials performed worldwide up to April 2021. In order to identify candidates for gene therapy in other neighboring countries we compared our findings with available literature from India and Pakistan which has utilized the same molecular diagnostic protocol to our study.

**Results:**

From the overall cohort of 623 rare disease patients with the characteristic clinical findings suspected of HD, SCA, SMA and Muscular Dystrophy, n = 343 (55%) [Muscular Dystrophy- 65%; (DMD-139, Becker Muscular Dystrophy -BMD-11), SCA type 1–3–53% (SCA1–61,SCA2- 23, SCA3- 39), HD- 52% (45) and SMA- 34% (22)] patients were positive for molecular diagnostics by MLPA and single plex PCR. A total of 147 patients in Sri Lanka amenable to available gene therapy; [DMD-83, SMA-15 and HD-49] were identified. A comparison of Sri Lankan finding with available literature from India and Pakistan identified a total of 1257 patients [DMD-1076, SMA- 57, and HD-124] from these three South Asian Countries as amenable for existing gene therapy trials. DMD, SMA, and HD gene therapy clinical trials (113 studies) performed worldwide up to April 2021 were concentrated mostly (99%) in High Income Countries (HIC) and Upper Middle-Income Countries (UMIC). However, studies on the potential use of anti-sense oligonucleotides (ASO) for treatment of SCAs have yet to reach clinical trials.

**Conclusion:**

Most genetic therapies for neurodegenerative and neuromuscular disorders have been evaluated for efficacy primarily in Western populations. No multicenter gene therapy clinical trial sites for DMD, SMA and HD in the South Asian region, leading to lack of knowledge on the safety and efficacy of such personalized therapies in other populations, including South Asians. By fostering collaboration between researchers, clinicians, patient advocacy groups, government and industry in gene therapy initiatives for the inherited-diseases community in the developing world would link the Global North and Global South and breathe life into the motto “Together we can make a difference”.

## 1 Introduction

**“What makes gene therapies different is that someone is going to be saying: I can fix this in your child forever and yet you can’t get it because it costs too much”-**

*Arthur Caplan, Professor of Bioethics at New York University Langone Medical Center - interview with Evaluate Vantage 2019.*

At a time where the world is to celebrate the 45th anniversary of the Alma Ata declaration to provide “health for all by the year 2000″ (Bhutta et al., 2018), the world’s priciest drug has been recently approved by the U. S. Food and Drug Administration (FDA) to treat SMA through a single-dose gene therapy priced at $2.125 million. Recently, an Indian couple in Hyderabad raised 16 crores in Indian rupees ($2.18 million) from nearly 65,000 donors to pay for a one-time gene therapy for their 3-year-old son with SMA. The one-time gene therapy is a competitor of the previously approved SMA treatment that one must continue for a lifetime at a yearly cost of $375,000, which would add up to 4.5 million USD in 10 years (Kerpel-Fronius et al., 2020). Similarly, the FDA-approved gene therapy for Duchenne muscular dystrophy (DMD) costs an average of $300,000 per patient annually (Shahryari et al., 2019). Up to date information on clinical trial phase and FDA/ European Medical Agency (EMA) approval status of available gene therapy for DMD, SMA and HD are summarized in Table 1.

**Table 1:** Clinical trial phase and FDA/ EMA approval status of available gene therapy for DMD, SMA and HD.

Therapy	Phase I	Phase II	Phase III	FDA/EMA approval	Reference
*Duchenne Muscular Dystrophy*					
Eteplirsen (exon 51 skipping)				√	(Syed, 2016)
Golodirsen (exon 53 skipping)				√	(Heo, 2020)
Viltolarsen (exon 53 skipping)				√	(Dhillon, 2020)
Casimersen (exon 45 skipping)				√	(Shirley, 2021)
Ataluren (stop codon readthrough)				√	(Huang et al., 2022)
Microdystrophin with AAV9 vector (gene addition)	√	√			(Duan, 2018)
*Spinal Muscular Atrophy*					
Nusinersen (antisense oligonucleotide)				√	(Hoy, 2019, Iftikhar et al., 2021)
Risdiplam (Small molecule)				√	(Dhillon, 2020, Kwon et al., 2022)
Onasemnogene abeparvovec (scAAV9 gene replacement)				√	(Hoy, 2019, Mahajan, 2019)
*Huntington’s Disease*					
rAAV5-miHTT	√	√			(Spronck et al., 2021)
rAAV1-(mi)RNA HTT	√				(Kim et al., 2021, Miniarikova et al., 2018)
WVE-120101 and WVE-120102	√	√			(Byun et al., 2022)

Nearly a quarter of the world's population live in the countries of South Asia, which include Afghanistan, Pakistan, India, Nepal, Bhutan, Bangladesh, the Maldives, and Sri Lanka. South Asia, a melting pot of genetic diversity, is home to thousands of ethnically and culturally diverse populations (Tätte et al., 2019). While consanguineous marriages are nearly uncommon or prohibited by societal pressure in Western societies, in many parts of Africa and Asia, including South Asia, approximately 20–50% of marriages are consanguineous (Mobarak et al., 2019). In Sri Lanka a study by Premawardhena et al., 2020 reveals that the overall national consanguinity rate is 7.4% and it was significantly higher among ethnic Tamils (22.4%) compared with Sinhalese (3.8%) and Moors (3.2%) (p < 0.001) (Premawardhena et al., 2020). Intriguingly Premawardhena et al., 2020 further highlight that when compared with the regional countries in South Asia, love marriages are becoming more popular in Sri Lanka where the decision on partner selection is likely to be made by the young people themselves, with attempts by parents to impose a partner choice very likely to be futile. This may be a reason for the observed less consanguinity rate reported in Sri Lanka compared to the regional countries in South Asia (Premawardhena et al., 2020). In India the overall prevalence of consanguineous marriage was 9.9%; the South region (23%) and North-East region (3.1%) showed the highest and lowest prevalence, respectively where Muslims had a higher prevalence (15%) than Hindus (9%). In Pakistan, and Bangladesh approximately 70% and 41% of marriages are consanguineous respectively, where in Bangladesh only 7% of couples have at least some knowledge about the potential genetic complications related to consanguinity (Anwar et al., 2020).

Intriguingly, it has been documented that approximately 85,000 years ago, the first wave of human migration out of Africa followed the coast through the Middle East into Southern Asia via Sri Lanka (Kundu and Ghosh, 2015). Most importantly, the only precursor of modern human fossils in South Asia, dating back 37,000 years, was found in Sri Lanka (Ranaweera et al., 2014).

We hypothesized that due to lack of comparative data available on potential candidates for available gene therapy clinical trials in DMD, SMA and HD, South Asia may have left behind in multicenter gene therapy clinical trials. In this article the authors discuss how they utilized the genetic mutation data in Sri Lankan DMD, SMA and HD patients and compare the findings with available literature from South Asian countries to identifying potential candidates for available gene therapy for DMD, SMA and HD patients.

## 2 Material and methods

### 2.1 Patient recruitment

Patients (n = 623) with the characteristic clinical findings suspected for HD, SCA, SMA and Muscular Dystrophy were recruited through Island wide neurology clinics, pro bono mobile clinics and home visits in selected government hospitals in Western, North-Western, North Central, Central, Southern Provinces and Northern Province in Sri Lanka from 2014 to 2022.

Patients who were clinically diagnosed by Consultant Neurologist/ Consultant Paediatric Neurologist were referred to the Interdisciplinary Center for Innovation in Biotechnology and Neuroscience (ICIBN), University of Sri Jayewardenepura, and Neuro-Molecular diagnostics and Neuro-Biobank, Institute for Combinatorial Advanced Research and Education (KDU-CARE), General Sir John Kotelawala Defence University (KDU), Sri Lanka for the genetic testing.

Written informed consents were obtained from every participant where applicable. For incompetent patients, surrogate consent was taken. This study meets the ethical guidelines of the Sri Lankan institutional review boards which follow the Helsinki Declaration (Ethical Approval Nos. 449/09, 38/19 and 34/14 from The Ethics Review Committee, Faculty of Medical Sciences, University of Sri Jayewardenepura and Ethical Approval No. LRH/D/06/2007 Lady Ridgeway Hospital for Children, Sri Lanka).

### 2.2 Molecular diagnostics

**Fig. 1:**
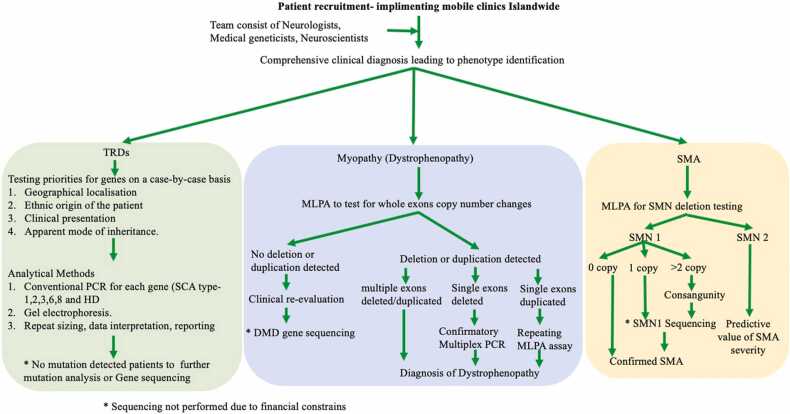
Molecular diagnostic approach.

For muscular dystrophies; DMD, Becker muscular dystrophy (BMD) and SMA, mutation detection protocols included Multiplex Ligation Dependent Probe Amplification (MLPA). HD and SCA molecular diagnostics were based on single plex PCR. Molecular diagnostic approach has been summarized in Fig. 1.

DMD and BMD molecular diagnostic protocols were based on the initial molecular diagnostic guidelines by Abbs et al., 2010 and the updated version by Fratter et al., 2020 (Abbs et al., 2010, Fratter et al., 2020). In summary, level one testing approach for deletions and duplications detection was followed, where MLPA assay (MRC Holland SALSA MLPA Probemixes P034 and P035) was applied as the initial diagnostic test. Since MLPA possesses the exon level of resolution for detection of deletions or duplications, the reliability of MLPA is high for Copy Number Variations extends for multiple adjacent exons, but less for single-exon deletion or duplication. Thereby in accordance to the European Molecular Quality Genetics Network (EMQN) best practice guidelines for genetic testing in dystrophinopathies, in our dystrophenopathy cohort, single exon deletions were re- confirmed by Multiplex PCR and single exon duplications were re-confirmed by repeating the MLPA assay. Molecular diagnostic protocol for SMA was based on the guidelines updated in 2020 (Prior et al., 2020) and diagnostic algorithm for SMA (Mercuri et al., 2018) utilizing MLPA assay (MRC Holland SALSA MLPA Probemixes P021 and P60).

SCA genetic analysis was also based on the EMQN guidelines (Sequeiros et al., 2010) where we have modified SCA testing priorities and considered testing for further genes on a case-by-case basis on the basis of local population disease frequencies, the geographic and ethnic origin of the patient and the information provided in the referral with regard to clinical presentation and the apparent mode of inheritance. HD genetic analysis was also based on the EMQN guidelines (Losekoot et al., 2013).

The accuracy, precision and validity of molecular diagnostics performed in our laboratory was confirmed by re-evaluating random samples of DMD, HD, SCA and SMA for genetic mutations following the same protocol at collaborative laboratories in India (Center for DNA Fingerprinting & Diagnostics, Institute of Genomics and Integrative Biology). Moreover at the inception the positive controls were provided by the Center for DNA Fingerprinting & Diagnostics, Institute of Genomics and Integrative Biology, India.

### 2.3 Clinical Trial Database assessment

A survey was conducted in The Wiley database on Gene Therapy Trials Worldwide (Medicine TJoG., 2021) to identify DMD, SMA, SCA and HD gene therapy clinical trials performed worldwide up to April 2021. To identify candidates for gene therapy in other neighboring countries we compared our findings with available literature from India and Pakistan which has utilized the same molecular diagnostic protocol to our study.

## 3 Results

Total of 623 patients with the characteristic clinical findings suspected for HD (n = 87), SCA (n = 234), SMA (n = 66) and Muscular Dystrophy (n = 236) were undergoing molecular diagnostics for the respective mutations according to the method summarized in Fig. 1. The sociodemographic data and molecular diagnostic results of these patients are tabulated in Table 2 & Table 3 respectively.

**Table 2:** Sociodemographic data of the patients with the characteristic clinical findings suspected of HD, SCA, SMA and Muscular Dystrophy who were referred to our molecular diagnostic facility.

Characteristic	Muscular Dystrophy (n = 236)	Spinocerebellar Ataxias (n = 234)	Huntington’s Disease (n = 87)	Spinal Muscular Atrophy (n = 66)
**Age range** (Mean)	1.5–18 (Mean-08Yrs).	21–73 (Mean-44Yrs).	25–59 (Mean-45 Yrs).	0.1–43 (Mean-33 Yrs)
**Gender** (Male: Female)	M-233:F- 03	M-126:F- 108	M-51:F-36	M-41:F- 25

**Table 3:** Molecular diagnostic summery of the patient cohort (Muscular Dystrophy, Huntington’s Disease, Spinal Muscular Atrophy, Spinocerebellar Ataxias).

Disease	Mutation	Sum of Count		%
**Huntington’s Disease**	Repeat expansion in HTT gene (HD)	45		52%
	No Repeat expansion in HTT gene	42		48%
**Huntington’s Disease Total**		**87**		
**Muscular Dystrophy**	Deletion or duplication detected in Dystrophin gene by MLPA (DMD)	139		59%
	Inframe Deletion or duplication detected in Dystrophin gene by MLPA (BMD)	11		5%
	Undergo genetic test by MLPA but no deletion or duplication detected in 79 exons in Dystrophin gene	86		36%
**Muscular Dystrophy Total**		**236**		
**Spinal Muscular Atrophy**	Undergo genetic test by MLPA but no deletion or duplication in SMN1 gene detected	44		67%
	Zero copies of SMN1 and 01 copy of SMN2 detected by MLPA (SMA type 0)	4		6%
	Zero copies of SMN1 and 02 copies of SMN2 detected by MLPA (SMA type 1)	4		6%
	Zero copies of SMN1 and 03 copies of SMN2 detected by MLPA (SMA type 2)	7		11%
	Zero copies of SMN1 and 3–4 copies of SMN2 detected by MLPA (SMA type 3)	6		9%
	Zero copies of SMN1 and > 4 copies of SMN2 detected by MLPA (SMA type 4)	1		2%
**Spinal Muscular Atrophy Total**		**66**		
**Spinocerebellar Ataxias**	Undergo genetic test for SCA 1,2,3,6,8 but no repeat expansion detected in respective genes	111		47%
	Repeat expansion in ATXN1 gene (SCA type 1)	61		26%
	Repeat expansion in ATXN2 gene (SCA type 2)	23		10%
	Repeat expansion in ATXN3 gene (SCA type 3)	39		17%
**Spinocerebellar Ataxias Total**		**234**		

From the overall cohort, 343/623 (55%) rare disease patients; [Muscular Dystrophy- 65%; (DMD n= 139, BMD n= 11), SCA type 1‐3 ‐ 53% (SCA1 n= 61,SCA2 n= 23, SCA3 n= 39), HD- 52% (n= 45) and SMA- 34% (n= 22)] were positive for initial molecular diagnostics by MLPA and single plex PCR. Among the genetically confirmed patients, DMD (60% n = 83/138), SMA type 0 - type II, and HD patients are identified as eligible to be considered for available gene therapy trials based on their mutation pattern (Table 5). Unfortunately, even though the mutation pattern of all the genetically confirmed SCA patients (n = 123) identified to be amenable for gene therapy, studies on the potential use of anti-sense oligonucleotides (ASO) for treatment of SCAs have yet to reach clinical trials. However, our results on SCA will showcase gene therapy eligible SCA patients on South Asian origin for future phase III clinical trials. Intriguingly in Sri Lankan and Indian DMD cohorts exon 51 skipping identifies the highest number of total patients eligible followed by exon 53 skipping, as presented in Table 5. When the availability of ongoing gene therapy clinical trials for DMD, HD, SMA and SCA were surveyed in the Wiley database on Gene Therapy Trials Worldwide, unfortunately no ongoing gene therapy clinical trials for SCA could be identified. While the available gene therapy trials for DMD, HD and SMA were considered, it was identified that, of the available 113 clinical trials, 104 (92%) are being performed in “High Income” countries where n = 8 (7%) trials are in “Upper Middle Income” countries as presented in Table 4.

**Table 4:** DMD, HD, SMA Gene therapy clinical trials worldwide.

Region	Country	World Bank country classification	DMD	SMA	HD
Europe	France	HI	6	1	2
	Germany	HI	4	2	1
	Netherlands	HI	4	2	0
	Norway	HI	2	0	0
	Switzerland	HI	0	0	1
	United Kingdom	HI	6	3	1
	Belgium	HI	3	3	0
	Denmark	HI	2	0	1
	Spain	HI	4	3	0
	Sweden	HI	0	1	0
	Bulgaria	UMI	1	0	0
	Russia	UMI	2	0	0
	Hungary	HI	1	0	0
	Italy	HI	4	3	0
	Poland	HI	1	0	1
North America	USA	HI	12	7	1
	Canada	HI	2	0	1
South America	Argentina	UMI	2	0	0
	Brazil	UMI	1	0	0
	Chile	HI	2	0	0
East Asia	Taiwan	HI	2	2	0
	South Korea	HI	1	2	0
	Japan	HI	2	1	0
	North Korea	LI	1	0	0
South Asia	-	-	0	0	0
Middle East	Israel	HI	2	1	0
	Turkey	UMI	2	0	0
Oceania	Australia	HI	2	1	1
Africa	-	-	0	0	0
**World Bank Country Classification**				**DMD, HD, SMA Gene therapy clinical trial**	
				**n**	**%**
Low-Income Economies ($1045 Or less) -LI				1	0.88
Lower-Middle Income Economies ($1046 To $4095)- LMI				0	0
Upper-Middle-Income Economies ($4096 To $12,695)- UMI				8	7.08
High-Income Economies ($12,696 Or More)- HI				104	92.04

**Table 5:** Patient cohort amenable for available Gene Therapy.

**DMD**				
Study	Sri Lanka, Present Study*	North India (Kohli et al., 2020)	Tamil Nadu, India (Kumar et al., 2020)	Pakistan (Ansar et al., 2019)
Total patients	139	1256	816	44
DMD Exon 51 Skipping	30 (21%)	211 (17%)	117 (14%)	7 (16%)
DMD Exon 53 Skipping	19 (13%)	152 (12%)	88 (11%)	9 (20%)
DMD Exon 45 Skipping	19 (13%)	129 (10%)	26 (3%)	7 (16%)
DMD Exon 50 Skipping	9 (6%)	44 (4%)	0	3 (7%)
DMD Exon 44 Skipping	5 (3%)	113 (9%)	15 (2%)	3 (7%)
DMD Nonsense Mutation	1 (0.7%)	34 (2.7%)	35 (4%)	N/A
Total patients amendable for gene therapy	**83 (60%)**	**964** (47%)		**29** (66%)

**TRDs**			
Study	Sri Lanka, Present Study*	Eastern India (Hussain et al., 2020)	Pakistan
Total patients	HD − 87	HD-75	N/A
HD ASO based therapy	49 (58%)	75	
Total patients amendable for gene therapy	**HD-49**	**HD-75**	

**SMA**				
Study	Sri Lanka, Present study*	South India (Swaminathan et al., 2008)	North India (Kesari et al., 2005)	Pakistan (Ibrahim et al., 2012)
Total patients	66	37	50	67
SMA	Type 0-04 Type I- 04 Type II- 07	Type I- 07 Type II- 04	Type I- 11 Type II- 05	Type I- 12 Type II- 03
Total patients (SMA Type 0-II) amendable for gene therapy	**15 (23%)**	**27 (31%)**		**15 (22%)**

* On going molecular genetic study by the corresponding author in Sri Lanka

## 4 Discussion

As indicated in Table 4, DMD, SMA and HD gene therapy clinical trials (113 studies) performed worldwide up to April 2021 were concentrated mostly (99%) in High Income Countries (HIC) and Upper Middle-Income Countries (UMIC). Sadly, only 11 studies were performed in Asia, but all were in the technologically advanced countries in East Asia. It is ironic and noteworthy that South Asians have been excluded from these clinical trials even though they constitute 40% of the Asian and 25% of the global population. Such exclusion may be mainly due to lack of established and or well-maintained patient registries in Global South including South Asian countries. In line with our analysis on DMD, SMA and HD gene therapy clinical trials, a survey of all gene therapy clinical trials reported on ClinicalTrials.gov as of November 22, 2021, reported by Cornetta et al., 2022 identified 171 recruiting trials (Cornetta et al., 2022). Intriguingly Cornetta et al., detailed that most of these gene therapy clinical trials were supported and established in HIC, often with multiple recruiting sites in HIC targeting more patients from HICs, where the authors hardly found any open trial or recruiting site in Lower Middle Income and Low-Income Countries.

In step with the World Health Organization’s vision of “harnessing genomic knowledge and having it contribute to health equity, especially among developing nations”, the corresponding author of this article received a donation of equipment through the International Brain Research Organization- Asia Pacific Regional Committee (IBRO-APRC) from National Institute of Neurological Disorders and Stroke (NINDS), of the National Institute of Health (NIH) USA in setting up,

1. the only free genetic testing service in the state sector for selected neurodegenerative and neuromuscular disorders in Sri Lanka, which currently provides free genetic testing reports (Samaranayake et al., 2020),
2. a DNA bank in a developing country, Sri Lanka, with samples from over 2000 patients with neurological disorders that include 623 samples from patients with rare diseases (Wijekoon et al., 2020),
3. A Brain Bank” - collections of 76 autopsy aging brain samples, performed with histopathological/immunohistochemical staining for neuropathology with genotyping for selective candidate genes, and
4. Human resource development through international training scholarships for postgraduates and Neuroscience workshops conducted in Sri Lanka with funding from IBRO- APRC. Moreover Double Doctoral Opportunities with international universities are available and ongoing.

Establishing a free molecular diagnostic service for selected Trinucleotide Repeat Disorders and neuromuscular disorders is a benchmark for countries in a region where genetic tests are almost unreachable in terms of availability and price. In India a free molecular diagnosing facility ( http://gomed.igib.in) has been established by the Institute of Genomics and Integrative Biology, New Delhi. It has been reported in the literature that in Pakistan, the cost of a PCR test for 22 exons of the DMD gene is approximately 333 USD (Naseem and Jawad, 2017), and in India, the same test costs approximately 52 USD (Srivastava et al., 2020). Moreover, in India, genetic testing for HD and SCA panels (types 1, 2, and 3) costs 35 USD and 79 USD, respectively. In contrast, in Sri Lanka, the authors utilized a pro bono molecular diagnostic approach inclusive of MLPA test and PCR-based molecular diagnostics, with 27–54 USD spent on chemicals and consumables per test. Such cost-effective approach could genetically confirm, 343/623 (55%) rare disease patients [Muscular Dystrophy- 65%, SCA type 1–3–53%, HD- 52% and SMA- 34%] as indicated in Table 2 and Table 3. The cost is mainly covered by funding obtained by the principal investigator through international and local funding agencies. Thus, there is an urgent call to philanthropists and nongovernmental organizations to facilitate in establishing pro bono molecular diagnostic services in developing countries with limited funds available (Wijekoon et al., 2020).

Intriguingly through our cost effective molecular diagnostic approach we could successfully identify a total of 147 patients amenable for available gene therapy [DMD-83, SMA-15 and HD-49] (Table 5). As reported in literature from different DMD populations, the current exon skipping therapies have the potential to benefit 30% of patients, of which exon 51 skipping can be used in 14%, exon 53 skipping applies to 10%, and exon 45 skipping applies to 9%, respectively. Results from our cohort and studies from India and Pakistan (Table 5) also follow the same pattern where more candidates are amenable for exon 51 (Our study- 21%, North India- 17%, Tamil Nadu- 14% and Pakistan- 16%) followed by exon 53 (Our study- 13%, North India- 12%, Tamil Nadu- 11% and Pakistan- 20%) and exon 45 (Our study- 13%, North India- 10%, Tamil Nadu- 03% and Pakistan- 16%) skipping (Aartsma‐Rus et al., 2009). We further compared our findings with available literature from India and Pakistan where we reported that a total of 1257 patients [DMD-1076, SMA- 57 and HD-124] could be identified from these three South Asian Countries- Sri Lanka, India and Pakistan as amenable for existing gene therapy trials (Table 5). However unfortunately these patients representing “Global South” are left behind from ongoing gene therapy clinical trials. This further highlights the health gap between rich and poor countries. Even though the Indian subcontinent, consisting of India, Nepal, Bangladesh, Pakistan, Bhutan, Sri Lanka, and the Maldives, is home to over 1.7 billion people (25% of the world population) (Kumar, 2019), there is insufficient knowledge and underrepresentation of scientific data on the prevalence and genetic subtypes these disorders. Thus, it is noteworthy that the few available scientific literature reports on these neurogenetic disorders are mainly from India and Pakistan where the representation is scanty for the other regional countries.

Pharmaceutical companies mainly justify the exorbitant costs of gene therapy via the high cost associated with the development, preclinical and clinical studies and manufacture (Hampson et al., 2018). However, it is noteworthy that most of the projects on developing gene therapies, especially for rare diseases have been initiated and accomplished utilizing public funds where the people in the society have indirectly contributed. Unfortunately this idea of public funds has not been taken into account when putting the price tag on the final product (Carr and Bradshaw, 2016, Bauer et al., 2017).

According to Allied Market Research (research Am, 2022), the global gene therapy market was worth “$6.0 billion in 2020 and is expected to reach $46.5 billion by 2030. Intriguingly the Asia-Pacific gene therapy market valued $349.1 million in 2020, which is projected to be $6931.5 Million in 2030. South Asians make up 25% of the global population, calling for pharma companies to consider ”which is better: Many customers at low price-point or few at a high price?” (research Am, 2022). Recent “World View” report by Anne Muigai (Muigai, 2022) in Nature Biotechnology further strengthen this idea, suggesting biotech industry to consider revising its business model and pricing strategy so it is more in line with those of blockbusters to improve the accessibility of therapies to patients across the globe, particularly those in developing nations.

Furthermore, according to the WHO, two billion people in emergent nations have limited access to vital medicines, further reinforcing the health gap between rich and poor countries (Ekström, 2021). Unfortunately, however, the World Trade Organization (WTO) intellectual property (IP) stance impedes the import, marketing, and production of generic versions of patented drugs (Ekström, 2021).

In this context, developing nations, led by India and South Africa, have proposed to the WTO that patents on vaccines and other COVID-19-related therapeutics should be waived, and this was further supported by the Director-General of the WHO in his timely and pointed statement, “Waive Covid vaccine patents to put the world on war footing”, an act that would be a stepping stone to addressing current health care inequities (Ghebreyesus, 2021).

**Fig. 2:**
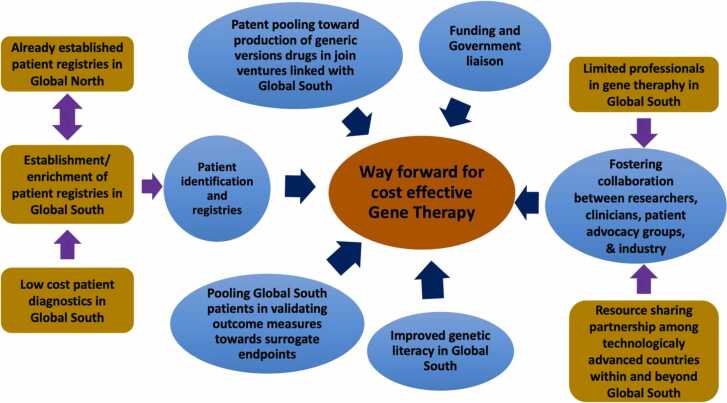
Way forward to implement low-cost gene therapy in “Global South”.

In this context, the authors would like to further suggest a model (Fig. 2) based on following urgent matters showcasing the potential way forward to implement low-cost gene therapy in “Global South”.

1. Issues with patient recruitment, attrition, comorbid conditions and expense have been identified as bottlenecks in designing long-duration clinical trials for rare diseases (Brooker et al., 2021). Therefore, when designing Phase 3 clinical trials for rare diseases, incorporating developing countries through multicenter studies is crucial.
2. As highlighted in literature, in DMD patients with deletion mutations, single exon skipping can cover approximately 70% of patients (Aartsma‐Rus et al., 2009), where it is reported that multiple exon skipping can theoretically be applied to approximately 90% of the patients. In this context using combinational antisense oligonucleotides in a cocktail is becoming the novel approach (Yokota et al., 2007, Echigoya and Yokota, 2014). The success of this approach depends on the comprehensive assessment of DMD genotype-phenotype associations in large cohorts. Thereby establishment and maintenance of global, regional and nationwide DMD databases is crucial (Deburgrave et al., 2007, Daoud et al., 2009, Bladen et al., 2015).
3. The untouched patient population in South Asia is a valuable resource for collaborative patient registries leading to future clinical trials and fluid biomarker (proteomic and metabolomic) identifications linking East and West.
4. Patent pooling (Bladen et al., 2015) and or waiving patents on gene therapy drugs and allowing developing countries to produce low-cost generics to serve the underprivileged would be a step toward mitigating current disparities in health care, especially among developing nations. In this context, the active contribution and leadership of the WHO and WTO are crucial. To this end, India is investing resources to become a cost-effective manufacturing hub for gene and cell therapy products (Cornetta et al., 2022).
5. It is noteworthy that Patients’ perceptions on gene therapy play a powerful role in implementing potential clinical trials. In this context ample number of evidence in literature highlight a general lack of genetic literacy, not only from patients but also from caregivers specially in developing countries (Tuckson et al., 2013, Vorderstrasse et al., 2014, Salgado et al., 2017, Strong et al., 2017), which largely contribute to inaccurate perception of gene therapy.

## 5 Conclusion

Most genetic therapies or personalized treatments for neurodegenerative and neuromuscular disorders have been evaluated for efficacy primarily in Western populations, and technologically advanced countries in East Asia. Hence, there is a lack of knowledge on the safety and efficacy of such personalized therapies in other populations, including South Asian patients. When multicenter gene therapy clinical trials for DMD, SMA and HD are planned, it is evident that trial sites from the South Asian region are not considered at all, as the nearest multicenter trial sites are in China and Taiwan. The substantial numbers of patients eligible for personalized therapies (India-1210, Sri Lanka-147, Pakistan-44) for DMD, SMA and HD in South Asia, warrant the inclusion in prospective multicenter clinical trials or study sites in Global South to ensure that no one is left behind, since these numbers are just the tip of the iceberg. By fostering collaboration between researchers, clinicians, patient advocacy groups, government, and industry in gene therapy initiatives for the inherited-diseases community in the developing world would link the Global North and Global South and breathe life into the motto “Together we can make a difference”.

## Compliance with Ethical Standards

This study meets the ethical guidelines of the Sri Lankan institutional review boards which follow the Helsinki Declaration (Ethical Approval Nos. 449/09, 38/19 and 34/14 from The Ethics Review Committee, Faculty of Medical Sciences, University of Sri Jayewardenepura and Ethical Approval No. LRH/D/06/2007 Lady Ridgeway Hospital for Children, Sri Lanka).

## Funding

The authors received funding from the 10.13039/100005202Muscular Dystrophy Association Washington DC, USA (grant number FMS/7090/2010), The World Health Organization (WHO) (grant number 2010/81594-0), the World Class University Grant Project (University of Sri Jayewardenepura, Sri Lanka; grant numbers WCUP/PhD/19 and WCUP/PhD/19B), the 10.13039/501100008640Ministry of Primary Industries, Sri Lanka (grant number SP/CIN/2016/02), the 10.13039/501100008816University of Sri Jayewardenepura (grant numbers ASP/06/RE/2010/07, ASP/06/RE/2012/18, ASP/06/RE/2013/28), General Sir John Kotelawala Defence University, Sri Lanka (grant numbers KDU/RG/2021/CARE/005 and KDU/RG/2021/CARE/006, and the Interdisciplinary Center for Innovation in Biotechnology and Neuroscience, University of Sri Jayewardenepura (ICIBN/ USJ). Equipment was donated by the National Institutes of Health (Bethesda, MD, USA) through IBRO-APRC and by the Chinese Neuroscience Society. Moreover, the corresponding author has received funding from IBRO-APRC and the International Society for Neurochemistry (10.13039/100009891ISN) for international training scholarships for postgraduates and to conduct Neuroscience workshops in Sri Lanka.

## CRediT authorship contribution statement

Nalaka Wijekoon and Lakmal Gonawala are Ph.D. scholars who contributed equally in establishing pro bono molecular diagnostic service by performing molecular diagnostic tests, genomic data interpretation, data collection through mobile clinics/ home visits and co-writing the manuscript. Pyara Ratnayake, Darshana Sirisena, Harsha Gunasekara, Athula Dissanayake, Sunethra Senanayake and Ajantha Keshavaraj are Consultant Neurologists/ Consultant Paediatric Neurologist and have contributed in performing the clinical diagnosis and evaluation of patients. Yetrib Hathout, and Chandra Mohan read, critically commented on the manuscript and accepted the final version. Harry W.M. Steinbusch, Ashwin Dalal, and Eric Hoffman are co-supervisors of Nalaka Wijekoon and Lakmal Gonawala who provided academic guidance, read, critically commented and accepted the manuscript. K Ranil D de Silva is the principal investigator of the project who pioneered with the concept of pro bono molecular diagnostic service and has developed the manuscript from its inception to the final version. All authors read and approved the final manuscript.

## Conflicts of Interest

The authors declare that they have no competing interests.
